# Inhibition of Mitogen-Activated Protein Kinase Erk1/2 Promotes Protein Degradation of ATP Binding Cassette Transporters A1 and G1 in CHO and HuH7 Cells

**DOI:** 10.1371/journal.pone.0062667

**Published:** 2013-04-25

**Authors:** Vishwaroop Mulay, Peta Wood, Melanie Manetsch, Masoud Darabi, Rose Cairns, Monira Hoque, Karen Cecilia Chan, Meritxell Reverter, Anna Álvarez-Guaita, Kerry-Anne Rye, Carles Rentero, Joerg Heeren, Carlos Enrich, Thomas Grewal

**Affiliations:** 1 Faculty of Pharmacy, University of Sydney, Sydney, New South Wales, Australia; 2 Departament de Biologia Cel·lular, Immunologia i Neurociències, Institut d’Investigacions Biomèdiques August Pi i Sunyer (IDIBAPS), Facultat de Medicina, Universitat de Barcelona, Barcelona, Spain; 3 Lipid Research Group, Heart Research Institute, Newtown, Sydney, New South Wales, Australia; 4 Faculty of Medicine, University of Sydney, Sydney, New South Wales, Australia; 5 Department of Biochemistry and Molecular Biology II: Molecular Cell Biology, University Medical Center Hamburg-Eppendorf, Hamburg, Germany; Loyola University Chicago, United States of America

## Abstract

Signal transduction modulates expression and activity of cholesterol transporters. We recently demonstrated that the Ras/mitogen-activated protein kinase (MAPK) signaling cascade regulates protein stability of Scavenger Receptor BI (SR-BI) through Proliferator Activator Receptor (PPARα) -dependent degradation pathways. In addition, MAPK (Mek/Erk 1/2) inhibition has been shown to influence liver X receptor (LXR) -inducible ATP Binding Cassette (ABC) transporter ABCA1 expression in macrophages. Here we investigated if Ras/MAPK signaling could alter expression and activity of ABCA1 and ABCG1 in steroidogenic and hepatic cell lines. We demonstrate that in Chinese Hamster Ovary (CHO) cells and human hepatic HuH7 cells, extracellular signal-regulated kinase 1/2 (Erk1/2) inhibition reduces PPARα-inducible ABCA1 protein levels, while ectopic expression of constitutively active H-Ras, K-Ras and MAPK/Erk kinase 1 (Mek1) increases ABCA1 protein expression, respectively. Furthermore, Mek1/2 inhibitors reduce ABCG1 protein levels in ABCG1 overexpressing CHO cells (CHO-ABCG1) and human embryonic kidney 293 (HEK293) cells treated with LXR agonist. This correlates with Mek1/2 inhibition reducing ABCG1 cell surface expression and decreasing cholesterol efflux onto High Density Lipoproteins (HDL). Real Time reverse transcriptase polymerase chain reaction (RT-PCR) and protein turnover studies reveal that Mek1/2 inhibitors do not target transcriptional regulation of ABCA1 and ABCG1, but promote ABCA1 and ABCG1 protein degradation in HuH7 and CHO cells, respectively. In line with published data from mouse macrophages, blocking Mek1/2 activity upregulates ABCA1 and ABCG1 protein levels in human THP1 macrophages, indicating opposite roles for the Ras/MAPK pathway in the regulation of ABC transporter activity in macrophages compared to steroidogenic and hepatic cell types. In summary, this study suggests that Ras/MAPK signaling modulates PPARα- and LXR-dependent protein degradation pathways in a cell-specific manner to regulate the expression levels of ABCA1 and ABCG1 transporters.

## Introduction

Anti-atherosclerotic properties of HDL and apolipoprotein A-I (apoA-I) include their ability to promote reverse cholesterol transport (RCT), the removal of excess cholesterol from peripheral tissues to the liver for bile secretion [1]–[3]. HDL receptors and ABC transporters are key molecules in cholesterol efflux from macrophages, with ABCA1 facilitating transfer of cholesterol onto apoA-I, while ABCG1 and SR-BI augment export of cholesterol onto HDL. In addition, ABCA1 in the liver is required for cholesterol export during HDL biogenesis, while hepatic SR-BI has a prominent role for the selective uptake of cholesteryl esters from HDL [1]–[3]. The molecular mechanisms of cholesterol transfer via ABC transporters and SR-BI have been studied extensively, however the signaling events that trigger mobilization of cellular cholesterol pools, or alternatively, alter expression and activity of cholesterol transporters are not fully understood. An increasing number of studies suggest that cell surface binding and internalization of HDL and apo-AI activate signaling proteins such as protein kinase A and C (PKA, PKC), Rac/Rho GTPases, Janus Kinase 2 (JAK2), calmodulin and MAPK to modulate the ability of cells to export cholesterol [4]–[6].

Given their potential as pharmaceutical targets, the control of ABC transporter and SR-BI expression received great attention, and transcriptional upregulation of ABCA1, ABCG1 and SR-BI via nuclear receptors, including LXR, PPARα and PPARγ, is well established [7], [8]. Yet, post-transcriptional mechanisms contribute to modify ABC transporters and SR-BI levels. Lysosomal as well as ubiquitin-dependent ABCA1 degradation implicated ABCA1 protein turnover as a modulator of cholesterol efflux [9]–[11]. In addition, ABCA1 contains a proline-glutamic acid-serine-threonine-rich (PEST) peptide sequence that accounts for calpain-mediated degradation along the lysosomal pathway [12]–[14]. Similarly, hepatic SR-BI protein levels are regulated post-transcriptionally by vitamin E, insulin, estrogen, the adaptor protein PDZ domain-containing protein 1 (PDZK1), as well as fibrates stimulating PPARα-dependent degradation pathways [15]–[18]. Little is known about ABCG1 protein turnover, but ubiquitination as well as calpain have recently been identified as influencing ABCG1 protein levels in macrophages [11], [19]–[21].

Activation of several signaling proteins, including PKC, PKA, Rac/Rho GTPases, JAK2 and calmodulin have been shown to affect ABCA1 and SR-BI protein stability [5]–[7]. Some signaling cascades are induced by HDL or apoA-I and linked to phosphorylation events targeting ABCA1, while others act via nuclear receptors and/or ubiquitination and proteosomal degradation pathways to modify ABCA1 and SR-BI levels [5]–[7], [13], [16]. In addition, we and others have demonstrated that Mek/Erk kinases contribute to alter ABCA1 and SR-BI expression and activity, most likely via nuclear receptors [22]–[25]. In lung epithelial cells, enhanced Erk1/2 signaling upregulates PPARα levels to increase ABCA1 mRNA expression and consequently, phospholipid efflux [22]. In macrophages, Erk1/2 inhibition protects LXR-induced ABCA1 mRNA from degradation to promote cholesterol efflux [23]. In contrast, in HepG2 cells Mek1/2 kinases act upstream of PPARγ- and LXRβ-dependent ABCA1 protein degradation [24]. Furthermore, we showed that inhibition of HDL-induced and SR-BI-mediated activation of the Ras/MAPK pathway [25]–[27] might establish feedback loops via PPARα to reduce SR-BI protein levels and activity in CHO cells and hepatic HuH7 cells [28]. In line with Erk1/2 kinases modulating nuclear receptor activity [29]–[31], HDL-inducible PPARα Ser21 phosphorylation was reduced in the presence of Mek1/2 inhibitor. Blocking Mek1/2 activity was associated with strongly decreased SR-BI cell surface expression and activity, as judged by reduced uptake of HDL-derived cholesteryl-esters [28].

Hence, differential regulation of ABCA1 and SR-BI protein levels in peripheral and hepatic cells by Mek/Erk kinases seem to involve multiple nuclear receptors. It is yet unknown if Mek1/2 inhibition can reduce ABCA1 protein levels in hepatic cells in a PPARα-dependent manner and if the Ras/MAPK pathway modulates protein levels of ABCG1. Here we demonstrate that Erk1/2 inhibition reduced ABCA1 protein stability in CHO and HuH7 hepatocarcinoma cells treated with PPARα agonists, while overexpression of constitutively active H-Ras, K-Ras or Mek1 increased ABCA1 expression. In addition, blocking Erk1/2 activity reduced ABCG1 protein levels in the presence and absence of LXR agonists in ABCG1 overexpressing CHO and HEK293 cells. This correlated with Erk1/2 inhibition strongly decreasing HDL-inducible cholesterol efflux in ABCG1 overexpressing CHO cells. Similar to Erk1/2 inhibition promoting SR-BI degradation in these cells [28], blocking MAPK activity reduced ABCA1 and ABCG1 protein stability without affecting ABC transporter mRNA expression. In contrast, Mek1/2 inhibitor PD98059 increased ABCA1 and ABCG1 protein levels in THP1 macrophages. In summary, depending on the cell-type and their repertoire of nuclear receptors, inhibition of the Ras/MAPK pathway could have opposite effects on ABCA1 and ABCG1 expression.

## Materials and Methods

### Reagents and Antibodies

Nutrient Mixture Ham’s F12, DMEM, RPMI-1640, geneticin, cycloheximide, fenofibrate (FF), Wy-14643, GW3965, T0901317, 12-O-tetradecanoylphorbol-13-acetate (TPA) were from Sigma. [^3^H]-Cholesterol was from Amersham Pharmacia Biotech. PD98059, U0126, CI-1040 were from Calbiochem. SDS-PAGE molecular weight markers were from Fermentas. Rabbit polyclonal antibodies against ABCA1, ABCG1 and SR-BI were from Novus. Rabbit polyclonal anti- PPARα was from Santa Cruz. Mouse monoclonal anti-Pan Ras and rabbit polyclonal anti-β-actin were from BD Transduction Laboratories. Rabbit polyclonal antibodies against activated Mek1/2 (P-Mek1/2), Erk1/2 (P-Erk1/2), Total Mek1/2, Total Erk1/2, glyceraldehyde 3-phosphate dehydrogenase (GAPDH) and Horseradish Peroxidase (HRP) -labeled secondary antibodies were purchased from Cell Signaling. Expression vectors encoding constitutively active H-Ras (HRasG12V), K-Ras (GFP-KRasG12V), Mek1 (Mek215-DD) and PPARα were kindly provided by John F. Hancock (Dallas, USA), Brian Gabrielli (Brisbane, Australia), and Bart Staels (Lille, France), respectively. CHO, THP1, HEK293 and HuH7 cell lines were from the American Type Culture Collection (ATCC, Manassas, VA, USA). CHO cells overexpressing ABCG1 (CHO-ABCG1) [19] were kindly provided by Ingrid Gelissen (Sydney, Australia). High Density Lipoproteins (HDL_3_, density 1.125–1.21 g/ml) were isolated from the plasma of normolipidemic volunteers by sequential ultracentrifugation in the 1.14< d <1.21 g/ml density range as described [28]. Purified apoA-I was prepared as described [32].

### Cell Culture

CHO wild-type (wt) and CHO overexpressing ABCG1 (CHO-ABCG1) were grown in Ham’s F12, HEK293 in DMEM, HuH7 in DMEM and F12 (1∶1), THP1 in RPMI-1640 together with 10% fetal calf serum (FCS), L-glutamine (2 mM), penicillin (100 U/ml) and streptomycin (100 µg/ml) at 37°C, 5% CO_2_. THP1 monocytes were differentiated with 2 nM TPA for 24 h before treatment with MEK inhibitors. For overexpression of HRasG12V, GFP-KRasG12V, Mek215-DD or PPARα, 1–2×10^5^ cells were transfected with 1.5 µg Qiagen-purified DNA and 6 µl of Lipofectamine 2000 (Invitrogen) as described [28].

### Real Time RT-PCR

Total RNA from HEK293 cells was extracted using the Trizol and RNeasy system (Macherey-Nagel, Germany) according to manufacturer’s instructions. 1 µg of RNA was reverse transcribed with the High Capacity cDNA Archive Kit (Applied Biosystems) and Real Time RT-PCR was performed as described previously [33]. Assay-on-Demand primer sets to amplify cDNA fragments encoding ABCA1, ABCG1 and TATA Box Binding Protein (TBP) sequences were from Applied Biosystems. Relative ABCA1 and ABCG1 expression was calculated by normalization to the housekeeper mRNA (TBP) as described [34].

### Cholesterol Efflux Assays

For the determination of cholesterol efflux, 2–5×10^5^ cells (in triplicate) were labeled overnight with [^3^H]-Cholesterol (2×10^6^ cpm/ml) as described [28]. Non-internalized radioactivity was removed by extensive washing with PBS. Then cells were incubated in Ham’s F12/0.1% BSA±50 µg/ml HDL_3_ (total protein) or 30 µg/ml apoA-I for 6 h, respectively. The media were harvested, cells were lysed in 0.1 N NaOH and the total cellular protein was determined [35]. The radioactivity in the media and cell lysate was measured by scintillation counting [28]. The ratio of released/(released and cell-associated) radioactivity×100 was calculated and is given in (%).

### Preparation of Cellular Extracts and Western Blot Analysis

Cells were harvested in lysis buffer (20 mM Tris-HCl, pH 7.5, 2 mM EDTA, 100 mM NaCl, 5 mM MgCl_2_, 1% (v/v) Triton X-100, 5 mM NaF, 10% (v/v) glycerol, 0.5% (v/v) 2-mercaptoethanol, 0.1 mM Na_3_VO_4_ and protease inhibitors). After centrifugation at 10000 g the protein concentration of the cleared cell lysate was determined. Cell lysates were separated by 10–12.5% SDS-PAGE and transferred to Immobilon-P (Millipore). Proteins were detected using their specific primary antibodies, followed by HRP-conjugated secondary antibodies and enhanced chemiluminescence detection (ECL, Amersham).

### PPARα Knockdown Studies

1–2×10^5^ HuH7 cells were transfected with 1.5 µg SureSilencing shRNA plasmids (SABiosciences) targeting human PPARα at Pos. 552–572 (5′-ggagcattgaacatcgaatgt-3′), 954–974 (5′-atgggtttataactcgtgaat-3′), and 1273–1293 (5′-tcaggaaaggccagtaacaat-3′) and Lipofectamine 2000 as described [28]. Studies were conducted after 72 h. Scrambled shRNA served as negative control (5′-ggaatctcattcgatgcatac-3′).

### Subcellular Fractionations

For the isolation of plasma membrane-enriched fractions, lysates from 1×10^7^ CHO-ABCG1 cells were separated on Percoll gradients as described [26], [28]. Cells were washed twice in 0.25 M Sucrose, 1 mM EDTA, 20 mM Tris-HCl, pH 7.8 plus protease inhibitors, collected and centrifuged. The postnuclear supernatant (PNS) was layered on top of 10 ml of 30% Percoll and centrifuged at 84.000 g for 30 min in a Beckman 70.1 TI rotor. The plasma membrane fraction in the middle of the gradient was isolated (1 ml), concentrated and analyzed for the amount of ABCG1 and Ras.

## Results

### Ras/MAPK Signaling Regulates ABCA1 Expression in CHO and HuH7 Cells

**Figure 1:**
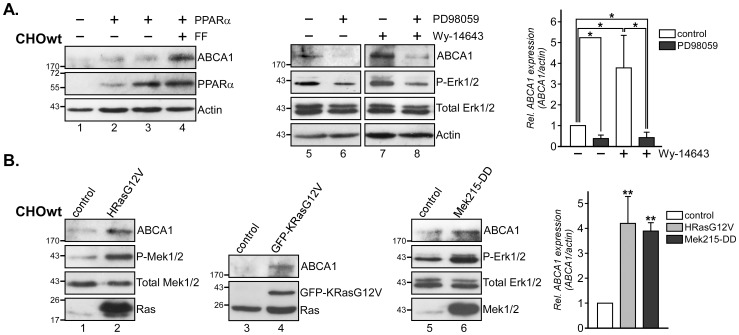
Ras/MAPK signaling regulates ABCA1 expression. (A+B) CHOwt cells were transfected with empty vector (−), PPARα (+), constitutively active H-Ras (HRasG12V), K-Ras (GFP-KRasG12V) or Mek1 (Mek215-DD) as indicated. (A) After 24 h cells were treated±20 µM Fenofibrate (FF), 20 µM Wy-14643 and±10 µM PD98059 as indicated. Cell lysates were analyzed for ABCA1, PPARα, activated Mek/Erk1/2 (P-Mek1/2, P-Erk1/2), Total Mek1/2 and Erk1/2, Ras and β-actin. Molecular weight markers are shown. ABCA1 levels were quantified, normalized to actin and represent the mean ± S.D. from 3 independent experiments. * and **, p<0.05 and p<0.01 for Student’s *t*-test, respectively.

Recent studies identified cell-type specific and differential involvement of Mek/Erk kinases in post-transcriptional pathways involving PPARγ and LXRα/β that regulate ABCA1 protein levels [23], [24]. In addition, we showed that Mek1/2 inhibition reduced PPARα-dependent SR-BI protein stability in CHO, HEK293 and hepatic HuH7 cells, but not peripheral cells, such as bovine aortic endothelial (BAEC) and monocytic (THP1) cell lines [28]. To determine if the Ras/MAPK pathway could alter ABCA1 protein levels, possibly in a PPARα-dependent manner, we first examined CHOwt cells, which have been proven a valuable model to study the involvement of ABCA1 in cholesterol transport [36], [37], exhibit HDL-inducible Ras/MAPK activity, Ras/MAPK-inducible PPARα phosphorylation and SR-BI expression [25]–[28]. CHOwt cells express low, but detectable levels of ABCA1 [36], as confirmed by western blot analysis using antibodies raised against human ABCA1 (see lane 1 in Fig. 1A and 1B). Similar to our previous studies, both moderate and high PPARα overexpression in CHOwt cells only modestly increased basal levels of its target genes in the absence of PPARα agonists, including SR-BI [28] and as shown here, ABCA1 (Fig. 1A, compare lane 1 with lane 2 and 3). Consistent with previous findings [7], [8], PPARα overexpression strongly stimulated ABCA1 expression in FF-treated CHOwt (Fig. 1A, lane 4). Wy-14643 (PPARα agonist) increased ABCA1 expression 3.8±1.4 fold in CHOwt cells (Fig. 1A, compare lane 5 and 7), and similar results were obtained in FF-treated CHOwt cells (not shown). Incubation with the Mek1/2 inhibitor PD98059 [38] reduced basal ABCA1 levels (compare lane 5 and 6) as well as Wy-14643 -induced ABCA1 levels by 89.4±4% (*, p = 0.039; Fig. 1A, compare lane 7 and 8). In contrast, ectopic expression of constitutively active HRas (HRasG12V), KRas (KRasG12V) and Mek1 (Mek215-DD) mutants in CHOwt cells increased ABCA1 protein levels 3.9 and 4.1 -fold, respectively (**, p = 0.0039; Fig. 1B). As HDL-induced Ras/MAPK signaling stimulates PPARα Ser21 phosphorylation in CHO cells [28], one can speculate that the Ras/MAPK pathway triggers phosphorylation events targeting PPARα that contribute to regulate ABCA1 expression in these cells.

**Figure 2:**
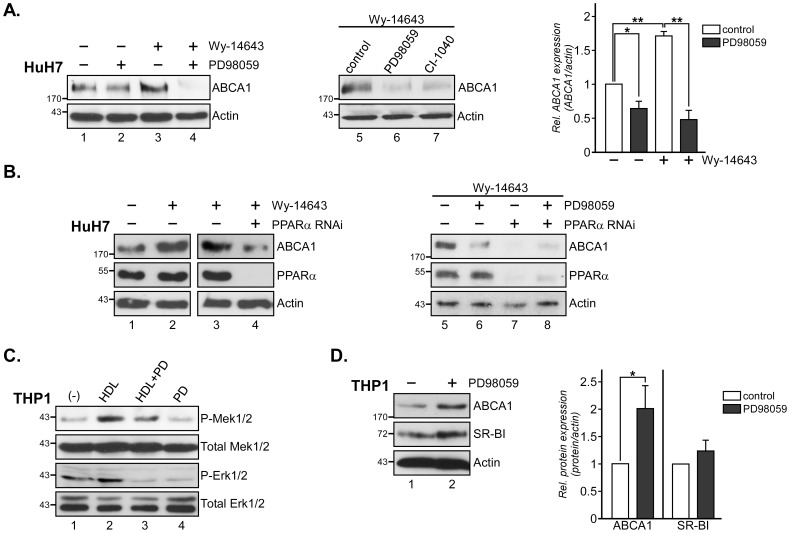
Mek1/2 inhibition reduces ABCA1 expression in HuH7 hepatocytes. (A) HuH7 cells were incubated for 24 h ±20 µM Wy-14643 with or without 10 µM PD98059 or CI-1040 as indicated. Cell lysates were analyzed by Western blotting for expression levels of ABCA1. ABCA1 levels in each lysate were quantified and normalized to the amount of β-actin. The mean values ± S.D. of 3 independent experiments are given. *and **, p<0.05 and p<0.01 for Student’s *t*-test, respectively. (B) HuH7 hepatocytes were transfected with control RNAi (−) or RNAi targeting PPARα (see Material and Methods). 72 h after transfection, cells were treated for an additional 24 h ±20 µM Wy-14643 and 10 µM PD98059 as indicated. Expression of ABCA1, PPARα, and β-actin was analyzed. Blots are representative for 2 independent experiments. (C) THP1 monocytes were differentiated with 2 nM TPA for 24 h, pre-incubated for 60 min ±10 µM PD98059 (PD), stimulated ± HDL_3_ (50 µg/ml) for 3 min and lysates were analyzed for activated Mek1/2 (P-Mek1/2), Erk1/2 (P-Erk1/2), Total Mek1/2 and Total Erk1/2. Blots are representative for 3 independent experiments. (D) TPA-differentiated THP1 monocytes were incubated ±10 µM PD98059 (PD) for 24 h as indicated. Western blot analysis of ABCA1, SR-BI and β-actin was quantified. *p<0.05 for Student’s *t*-test. Molecular weight markers are shown.

ABCA1 is important for hepatic HDL biogenesis, and we next investigated if similar mechanisms exist in hepatic HuH7 cells (Fig. 2A). Indeed, Mek1/2 inhibition in these cells reduced ABCA1 levels in the absence of Wy-14643 by 35.9±9.4% (*, p = 0.002; Fig. 2A, compare lane 1 and 2). Treatment of HuH7 cells with PPARα agonist induced ABCA1 expression 1.7±0.3 -fold (**, p = 0.0027; compare lane 1 and 3), which was drastically reduced by 77.9±8.1% in the presence of PD98059 (**, p = 0.0031; compare lane 3 and 4). The second generation Mek1/2 inhibitor CI-1040 (PD184352) [39] also reduced ABCA1 expression in Wy-14643 stimulated HuH7 cells (compare lane 5–7).

To substantiate the involvement of PPARα and Mek1/2 in ABCA1 expression, HuH7 cells were transfected with siRNA to knockdown endogenous PPARα (Fig. 2B). As shown above, Wy-14643 treatment of HuH7 cells transfected with control siRNA induced ABCA1 protein levels (Fig. 2B, compare lane 1 and 2). PPARα knockdown (≥95%) correlated with strongly reduced ABCA1 levels in Wy-14643 incubated cells (compare lane 3 and 4). Similar to Fig. 2A, PD98059 strongly abrogated ABCA1 expression in Wy-14643 stimulated cells (compare lane 5 and 6). In PPARα–depleted and Wy-14643-treated HuH7 cells, ABCA1 expression was strongly downregulated in the presence or absence of Mek1/2 inhibitor (lane 7–8). ABCA1 downregulation was less severe in PPARα-depleted cells in the presence of PD98059 (compare lane 7 and 8). This could be due to incomplete PPARα knockdown/inhibition or indicate that Mek1/2 inhibition in HuH7 hepatocytes modulates ABCA1 expression probably not exclusively via PPARα, but also via other nuclear factors, possibly PPARγ ανδ LXRβ, as shown for HepG2 cells [24].

In contrast to the results obtained from CHO and HuH7 cells (see above), Mek1/2 inhibition increases ABCA1 expression in RAW and mouse peritoneal macrophages in a dose-dependent manner [23]. Mek1/2 activation in macrophages can occur through various signaling pathways. HDL-induced activation of Mek/Erk kinases has been demonstrated in various cell types [25]–[28], [40]–[42] and Mek/Erk phosphorylation upon HDL incubation in TPA-differentiated THP1 monocytes indicates that this signaling cascade also exists in human macrophages (Fig. 2C, compare lane 1 and 2). HDL-induced Mek/Erk phosphorylation in THP1 macrophages is effectively inhibited by PD98059 (compare lane 2 and 3). To determine if Mek/Erk inhibition could also elevate ABCA1 expression in a human macrophage model system, THP1 monocytes were differentiated with TPA for 24 h before treatment ±10 µM PD98059 (Fig. 2D). Mek1/2 inhibition increased ABCA1 protein expression approximately 2-fold in these cells (*, p = 0.032; compare lane 1 and 2), which is comparable to data using similar PD98059 concentrations in RAW macrophages [23]. Consistent with our previous data [28], PD98059-incubated THP1 macrophages also showed a small, but not significant increase of SR-BI expression compared to controls (Fig. 2D). It remains to be determined if HDL-induced Erk1/2 activation contributes to modulate cholesterol efflux via controlling ABCA1 levels in macrophages *in vivo*, however findings from the hepatic HuH7 cells and THP1 macrophage model support opposite and cell-specific roles for Mek/Erk kinases in the regulation of ABCA1 expression.

### Mek1/2 Inhibition Reduces ABCG1 Expression in CHO Cells

**Figure 3:**
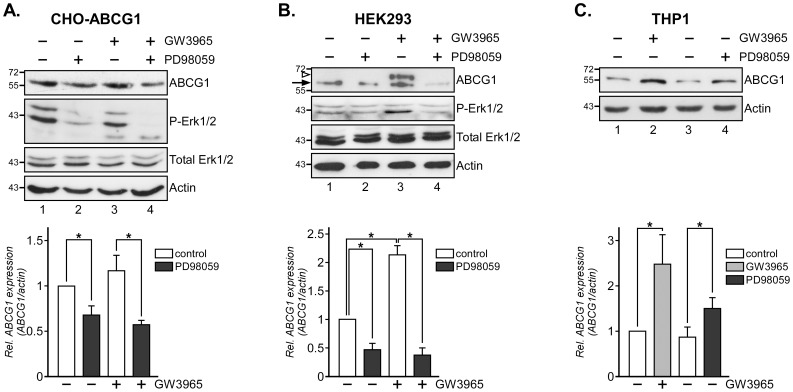
Ras/MAPK inhibition reduces ABCG1 expression in CHO cells. (A–C) CHO-ABCG1 (A), HEK293 (B) and THP1 macrophages (C) cells were incubated ±50 µM GW3965 and 10 µM PD98059 for 24 h as indicated. Cell lysates were analyzed for ABCG1, activated Erk1/2 (P-Erk1/2), Total Erk1/2 and β-actin. ABCG1 levels (see arrow) were quantified and normalized to β-actin. The arrowhead points at an additional GW3685-inducible and PD98059-sensitive protein (lane 3). Representative western blots for 3 independent experiments per cell line are shown. Molecular weight markers are shown. *, p<0.05 for Student’s *t* test.

Based on the Ras/MAPK pathway modulating SR-BI [28] and ABCA1 (Fig. 1–2) expression, we speculated that Mek1/2 inhibition could also modify the protein levels of ABCG1, another ABC transporter implicated in HDL cholesterol transport. Given LXR-inducible ABCG1 expression [1]–[3], [9], Mek kinases acting upstream of LXR in HepG2 cells [24], and the very low amounts of endogenous ABCG1 in CHOwt cells [43], we first utilized CHO cells stably overexpressing ABCG1 (CHO-ABCG1) [43] treated ± LXR agonist (GW3965) to determine if Mek1/2 inhibition could reduce ABCG1 levels. Indeed, PD98059 reduced ABCG1 protein levels by 33±10 and 52±5% (*, p = 0.022 and 0.035; Fig. 3A, compare lane 1 and 2, 3 and 4) in CHO-ABCG1 cells incubated ± GW3965, respectively. Endogenous ABCG1 levels in HEK293 cells, a common model to study cholesterol transport, ABCA1-dependent signaling and nuclear receptor activity [4], [28], [31], treated ± GW3965 were also reduced by approximately 40–70% with PD98059 (*, p = 0.040, 0.035 and 0.026; Fig. 3B, compare lane 1 and 2, 3 and 4). Using a commercial rabbit polyclonal ABCG1 antibody in this western blot analysis, we also observed an additional GW3685-inducible and PD98059-sensitive protein (see arrowhead, lane 3). These findings may indicate limitations of the human HEK293 model for ABCG1 expression studies when using rabbit polyclonal ABCG1 antibodies. Although we cannot completely rule out an unspecific signal not related to ABCG1, this might possibly indicate post-translational modification or expression of another ABCG1 isoform [19]. Further supporting an opposite role for Mek/Erk kinases in ABC transporter expression in macrophages, ABCG1 levels increased 1.4–1.6–fold in PD98059-incubated THP1 macrophages (*, p = 0.041; Fig. 3C, compare lane 1, 3 and 4). Co-incubation of LXR agonist and PD98059 did not further increase ABCG1 levels (not shown). Taken together, MAPK inhibition reduces ABCA1 (Fig. 1A, 2A–B), ABCG1 (Fig. 3A–B) and SR-BI [28] levels in CHO, HuH7 and HEK293 cells. In contrast, Mek1/2 inhibitor PD98059 increases ABCA1 (Fig. 2D), ABCG1 (Fig. 3D) expression and modestly elevated SR-BI protein levels (Fig. 2D) in THP1 macrophages.

### Blocking Mek1/2 Activity Reduces ABCG1-mediated Cholesterol Efflux in CHO Cells

**Figure 4:**
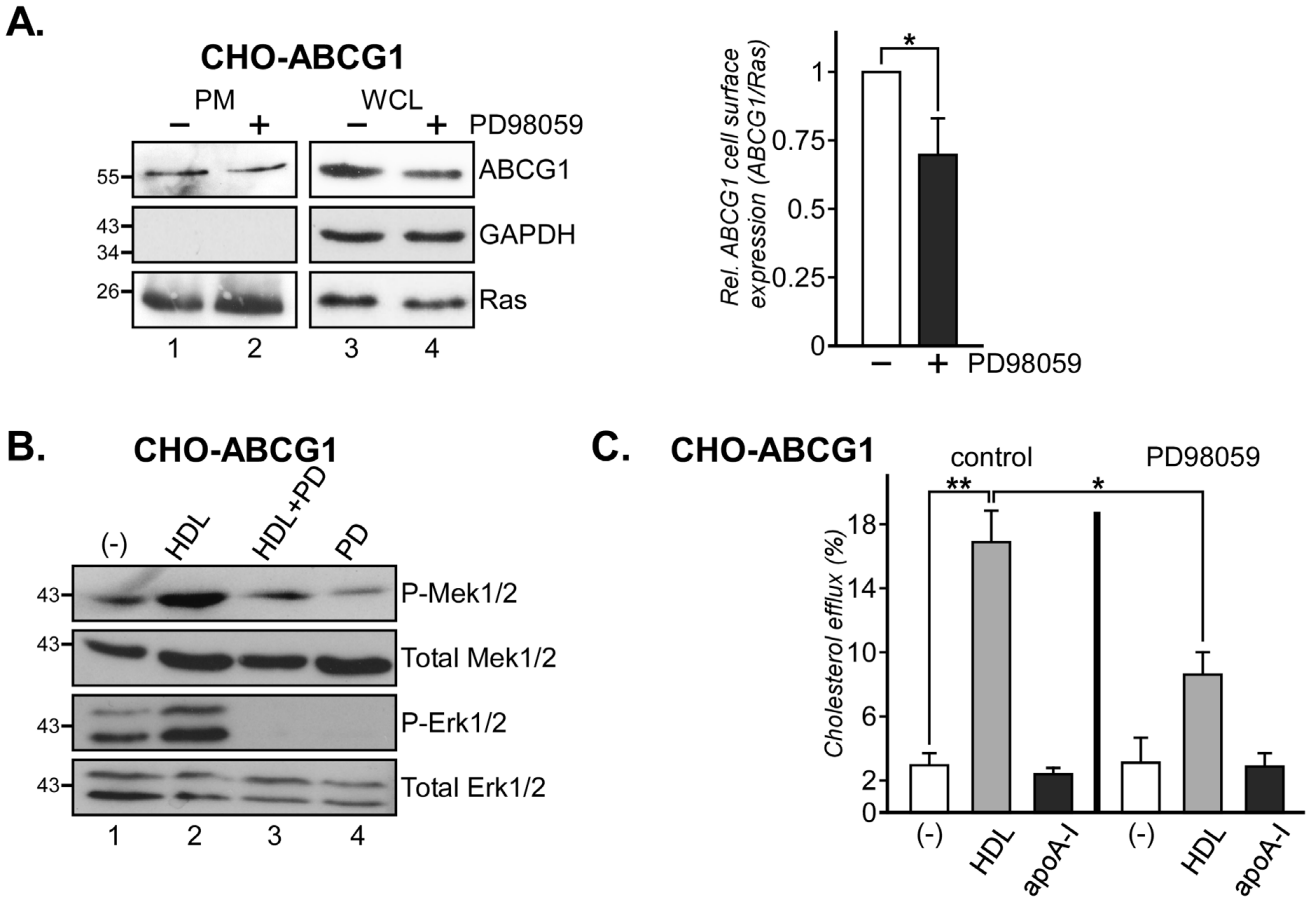
Ras/MAPK signaling modulates ABCG1 activity in CHO-ABCG1 cells. (A) CHO-ABCG1 cells were incubated ±10 µM PD98059 for 24 h as indicated and subjected to subcellular fractionation through Percoll gradients. Ras-containing plasma membrane fractions (PM) in the middle of the gradient were isolated and analyzed for the amount of ABCG1, and markers for cytosol (GAPDH) and plasma membrane (Ras). For comparison, western blot analysis of ABCG1, GAPDH (cytosol) and Ras from whole cell lysates (WCL) is shown. (B) CHO-ABCG1 cells were starved, pre-incubated for 60 min ±10 µM PD98059 (PD) and stimulated ± HDL_3_ (50 µg/ml) for 3 min at 37°C as indicated. Western blot analysis of activated Mek1/2 (P-Mek1/2), Erk1/2 (P-Erk1/2), Total Mek1/2 and Erk1/2 from each lysate is shown. (C) CHO-ABCG1 cells were incubated with [^3^H]-Cholesterol (2×10^6^ cpm/ml) for 24 h, washed with PBS, and incubated with HDL_3_ (50 µg/ml) or apoA-I (30 µg/ml) for 6 h±10 µM PD98059. The ratio of released and cell-associated radioactivity was determined, normalized to total cell protein and the amount of efflux is given in (%). The background efflux obtained from CHOwt was equivalent to 1.5–3.0×10^5^ cpm/mg cell protein. *and **, p<0.05 and p<0.01 for Student’s *t* test.

Erk1/2 inhibition has been shown to affect both ABCA1 expression and activity [22], [23], so we next aimed to identify if reduced ABCG1 levels in PD98059-treated CHO-ABCG1 cells would correlate with diminished ABCG1 transporter activity. We first determined ABCG1 cell surface expression. Western blot analysis of Ras-containing plasma membrane fractions isolated from Percoll gradients identified decreased ABCG1 levels in CHO-ABCG1 cells treated with PD98059 (*, p = 0.028; Fig. 4A). Next we examined the ability of PD98059 to inhibit HDL-inducible Ras/MAPK activation and cholesterol efflux in ABCG1 overexpressing CHO cells. As shown for CHOwt and CHO cells overexpressing SR-BI [28], HDL strongly activates Mek1/2 and Erk1/2 kinases (P-Mek1/2, P-Erk1/2) in CHO-ABCG1 cells (Fig. 4B, compare lane 1 and 2). HDL-induced Mek1/2 and Erk1/2 phosphorylation (compare lane 2 and 3) in these cells was inhibited with PD98059, while phosphorylation of other Ras effectors, such as Akt, was not altered (not shown). We then analyzed HDL-inducible cholesterol efflux in CHO-ABCG1 cells ± PD98059 (Fig. 4C). CHO-ABCG1 cells were labeled for 24 hours with [^3^H]-Cholesterol, pre-incubated for 4 h±10 µM PD98059, followed by an incubation with 50 µg/ml HDL or 30 µg/ml apoA-I for 6 h. Cells and media were assayed for radioactivity and efflux was determined as the percentage of total cholesterol in the culture. As shown previously [28], PD98059 alone did not affect [^3^H]-cholesterol internalization, cell viability or basal cholesterol efflux, nor did apoA-I activate the Ras/MAPK pathway in CHOwt cells [26] (not shown). Furthermore, treatment with PD98059 alone, and consistent with previous studies in CHO cells [43], incubation with apoA-I (30 µg/ml) ± PD98059 did not alter basal cholesterol efflux activities. In contrast, HDL effectively stimulated cholesterol efflux in CHO-ABCG1 cells (**, p = 0.0017; 5.6±0.7–fold). Yet, efflux of [^3^H]-cholesterol-loaded CHO-ABCG1 cells onto HDL in the presence of PD98059 was reduced by 53±11% (*, p = 0.012). Hence, the Ras/MAPK activity modulates ABCG1 expression and activity in CHO-ABCG1 cells.

### Ras/MAPK Signaling Modulates ABCA1 and ABCG1 Protein Stability

**Figure 5:**
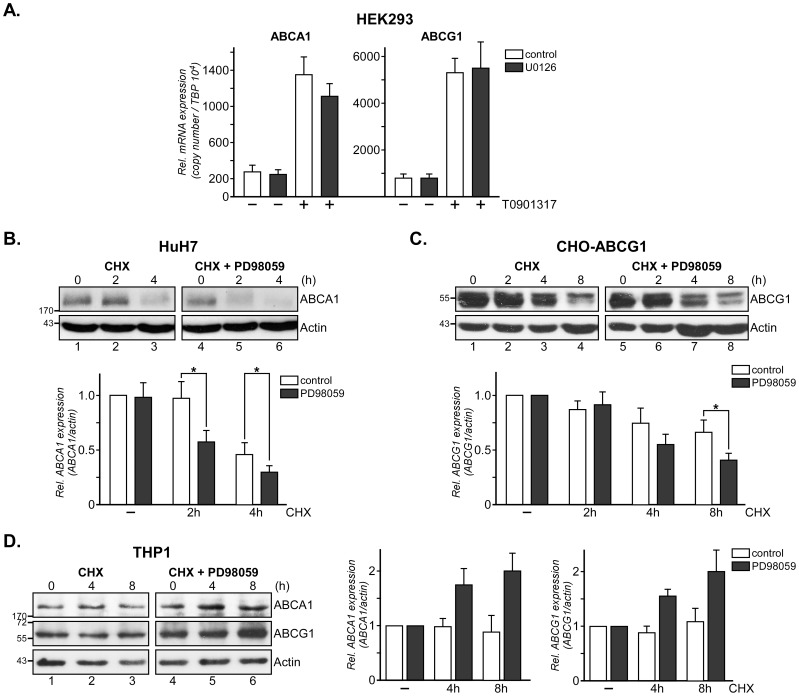
Mek1/2 inhibition reduces ABCA1 and ABCG1 protein stability. (A) 1 µg of RNA extracted from HEK293 cells treated ± LXR agonist (1 µM T0901317) and Mek1/2 inhibitor (10 µM U0126) was reverse transcribed and Real Time RT-PCR to amplify ABCA1, ABCG1, and TATA Box Binding Protein (TBP) cDNA fragments was performed as described [28]. Relative expression from 2 independent experiments with duplicate samples is given and was calculated by normalization to the housekeeper mRNA (TBP). (B–D) HuH7 (B) and CHO-ABCG1 (C) treated with 20 µM Wy-14643 or 50 µM GW53965 overnight and PMA-differentiated THP1 monocytes (D) were incubated with 20 ng/ml cycloheximide (CHX)±10 µM PD98059 (PD) for 0–4 (B) and 0–8 h (C, D) as indicated. Western blot analysis of ABCA1, ABCG1 and β-actin in each lysate of a representative experiment is shown. The mean values ± S.D. of ABCA1 and ABCG1 expression levels (n = 2) are given. *, p<0.05 for Student’s *t* test.

In CHO, HEK293 and HuH7 cells, Mek1/2 inhibition enhanced SR-BI protein degradation while SR-BI mRNA levels remained unchanged [28]. To identify if Ras/MAPK signaling modifies ABCA1 and ABCG1 mRNA or protein expression, we first measured ABCA1 and ABCG1 mRNA ± U0126, another specific Mek1/2 inhibitor [44], by Real Time RT-PCR in HEK293 cells (Fig. 5A). While Mek inhibition reduced ABCA1 and ABCG1 protein levels in unstimulated cells (Fig. 2A, 3A–B), mRNA levels of unstimulated HEK293±U0126 remained unchanged. As expected, treatment with LXR agonist (1 µM T0901317) increased ABCA1 and ABCG1 mRNA levels approximately 5 and 6–fold, respectively. However, co-incubation with U0126 did not significantly reduce ABCA1 or ABCG1 mRNA expression (Fig. 5A;+T0901317, compare white and black columns). Similarly, mRNA levels of ABCA1, ABCG1 and SR-BI remained unchanged upon Mek1/2 inhibition in primary mouse hepatocytes treated with the second generation Mek1/2 inhibitor CI-1040 [39] (not shown).

Finally we compared ABCA1 and ABCG1 protein stability, which both have a short half-life and are rapidly degraded [10], [19], in HuH7, CHO-ABCG1 and THP1 cells, respectively (Fig. 5B–D). To examine ABCA1 protein turnover, HuH7 cells were incubated with 20 ng/ml cycloheximide to inhibit protein synthesis for 0–4 h ± Mek1/2 inhibitor PD98059 (Fig. 5B). ABCA1 levels remained constant over 2 h and were strongly reduced after 4 h in controls (compare lane 1–3, see white columns for quantification). In contrast, accelerated ABCA1 protein degradation in the presence of PD98059 was evident already after 2 h, with ABCA1 protein levels being reduced by 45.6±8.6% (*, p = 0.010 and 0.038; lane 4–6, see black columns for quantification).

Primary hepatocytes and hepatic HuH7 cells express very little ABCG1, making it difficult to quantify ABCG1 protein degradation in these cells by western blotting. Therefore, to examine ABCG1 turnover, ABCG1 overexpressing CHO-ABCG1 cells were treated with cycloheximide ± Mek1/2 inhibitor PD98059 and ABCG1 levels were monitored for 0–8 h (Fig. 5C). After 4 and 8 h, ABCG1 protein levels in cycloheximide-treated CHO-ABCG1 cells were reduced by 35.1±7.3 and 33.5±13.3%, respectively (compare lane 1–4, white columns for quantification). In the presence of PD98059, ABCG1 degradation was more pronounced after 8 h (54.4±5.0%, black columns).

In line with Mek1/2 inhibition increasing ABCA1 and ABCG1 protein levels in THP1 cells (Fig. 2D, 3C), addition of Mek1/2 inhibitors to cycloheximide-treated THP1 cells slightly increased ABCA1 and ABCG1 protein stability by 1.4–2.0 -fold after 4–8 h, respectively (Fig. 5D, compare lanes 1–3 with 4–6). These findings possibly suggest opposite effects of Erk1/2 inhibition on degradation pathways that regulate ABCA1 and ABCG1 protein levels in HuH7 and CHO cells (Fig. 5B–C) compared to THP1 cells. Erk1/2 inhibition protecting ABCA1 mRNA degradation in macrophages, as shown by others [23], could further add to increase ABC transporter expression in PD98059-incubated THP1 cells. Taken together, Mek/Erk kinase inhibition is a common module to reduce not only SR-BI [28], but also ABCA1 and ABCG1 protein stability in HuH7 and CHO cells.

## Discussion

In this study we demonstrate that Ras/MAPK inhibition reduced protein levels of ABCA1 and ABCG1 in CHO, HEK293 and HuH7 cells, possibly via nuclear receptor (PPARα, LXR) -inducible degradation pathways. In these cells, Ras/MAPK signaling modulates ABCA1 and ABCG1 cell surface expression and activity. In contrast, blocking Mek1/2 activity increased ABCA1 and ABCG1 expression in THP1 macrophages. Thus, cell-specific regulation of ABCA1 and ABCG1 protein stability and cell surface expression via the Ras/MAPK signaling cascade could contribute to fine-tune reverse cholesterol transport.

The signal transduction pathways that participate in the regulation of ABC transporters are complex and poorly understood [4]–[6]. Several kinases, including PKC and PKA, directly phosphorylate ABC transporters. PKCα phosphorylates threonine residues T1286 and T1305 within the C-terminal PEST motif of ABCA1 to regulate calpain-dependent ABCA1 degradation [14], [45]. Another PKC family member, PKCδ, destabilizes ABCA1 via increased ABCA1 serine phosphorylation [46]. In addition, PKA-mediated phosphorylation of serine residues S2054 and S1042 stimulates ABCA1-mediated cholesterol efflux onto apoA-I [47], [48]. Besides PKC and PKA, protein kinase CK2 has also been implicated to phosphorylate and modulate ABCA1 activity [49]. With respect to ABCG1, recent studies identified serine, threonine and tyrosine phosphorylation of ABCG1 [20], as well as p38MAPK and JNK2-dependent pathways to promote ABCG1 serine phosphorylation and degradation [50].

To date, Mek and Erk kinases have not been identified to directly phosphorylate ABCA1 and ABCG1. As described above, Mek/Erk kinases rather seem to regulate ABCA1 and ABCG1 expression via nuclear receptors [22]–[24], [28]. Mek/Erk kinases can control nuclear receptors through multifaceted pathways including transcriptional and post-transcriptional regulation of PPARα/γ and LXR [22], [23], [29], [51], PPAR/LXR target gene mRNA stability [23] and PPARα/γ ligand production [52], as well as nuclear translocation of PPARs [53]. Recruitment of PPAR/LXR co-activators/repressors also has to be considered as Erk1/2 modulates co-repressor nuclear receptor co-repressor 1 (NCoR) activity [54] and phosphorylates retinoic acid receptors (RXRs), which impairs co-activator recruitment [55].

We previously demonstrated that enhanced Ras/MAPK signaling in CHO and HEK293 cells did not alter PPARα expression or localization, but increased Ser21-PPARα phosphorylation [28]. Erk1/2 phosphorylation events targeting PPARs are well documented [29], [30] and both PPARα and PPARγ contain MAPK phosphorylation sites that modulate their transcriptional activity. Erk1/2-mediated transactivation of PPARα occurs at serine residues S12 and S21 and PD98059 treatment blocks PPARα activity [29], [30]. In contrast, Erk2-mediated phosphorylation of S82 inhibits PPARγ1 transcriptional activity [29], [30]. These findings were predominantly derived from studies related to insulin signaling, but we showed that PD98059 interferes with HDL–induced S21-PPARα phosphorylation [28], indicating similar events during HDL-mediated signal transduction and RCT. Also, HDL-induced and MAPK-mediated phosphorylation of PPARγ in RAW macrophages is associated with reduced expression of PPARγ-responsive genes [53]. Most relevant to RCT, Erk1/2 inhibition correlated with decreased PPARγ phosphorylation *in vivo*, which was associated with increased PPARγ activity, elevated ABCA1 expression and reduced size of atherosclerotic lesions [56].

It remains to be determined how Mek1/2 inhibition alters ABCG1 protein expression in the presence of LXR agonists. In macrophages, Erk1/2 inhibition synergizes with LXR activation to induce ABCA1 expression [23]. Alternatively, in HepG2 cells it was proposed that Mek1/2 inhibition might interfere with direct interaction of LXRβ with ABCA1 [24]. Similar mechanisms might exist in the CHO and HEK293 models analyzed in this study. However, although LXRα becomes phosphorylated at a MAPK consensus site at S198, the physiological relevance of this phosphorylation is still unclear [31].

We previously showed that Mek1/2 inhibition promotes proteasomal degradation of SR-BI [28]. Interestingly, ubiquitin-dependent ABCA1 and ABCG1 degradation has also been observed [10], [11]. This degradation pathway is commonly initiated by serine/threonine phosphorylation or dephosphorylation events to recruit ubiquitin ligases, followed by ubiquitination of the target protein. Depending on the cell type, the same phosphorylation/dephosphorylation event can activate or inhibit the ubiquitin-proteasome machinery [57], hence providing opportunities for Mek/Erk kinases to prevent or enhance ABCA1, ABCG1 and SR-BI protein turnover in a cell-specific manner. Along these lines, comparison of gene arrays from livers of fibrate-treated wildtype and PPARα ko-mice and monkeys identified ∼30 upregulated PPARα target genes encoding for proteasome subunits and ubiquitin-activating/conjugating enzymes [58]–[60]. Also, the E2-conjugating enzyme Ubc9 and Sumo E3 ligase PIASγ promote recruitment of co-repressor NCoR to PPARα in HuH7 cells [61]. Hence, nuclear receptor-dependent pathways seem to affect multiple proteins involved in the machinery that controls the protein turnover of ABC transporters.

The diverse action of phosphorylation events and nuclear receptors on the multifactorial proteasome machinery adds to the complexity of the physiological relevance of signal transduction targeting ABC transporters and HDL receptors in RCT. Since the key findings of our study were derived from various cell culture models, future *in vivo* studies using mouse models will have to substantiate a role for the Ras/MAPK pathway in modulating protein stability of ABC transporters, HDL metabolism and RCT. We speculate that HDL-induced activation of Ras/MAPK could be an opportunity to fine-tune the activity and contribution of PPARα, PPARγ and LXR in peripheral and hepatic ABC transporters and SR-BI expression. Identifying the physiological stimuli that enable Mek/Erk kinases to differentially regulate the protein stability of ABC transporters and SR-BI may provide insights into the contribution of growth factors, insulin and hormones for hepatic ABCA1 and SR-BI activity and deliver clues to explain how Ras/MAPK overactivation contributes to atherosclerotic lesion development [62]–[64].
